# The relationship between general practice characteristics and quality of care: a national survey of quality indicators used in the UK Quality and Outcomes Framework, 2004–5

**DOI:** 10.1186/1471-2296-7-68

**Published:** 2006-11-13

**Authors:** Mark Ashworth, David Armstrong

**Affiliations:** 1 Department of General Practice & Primary Care, King's College London School of Medicine at Guy's, King's College and St Thomas' Hospitals, London, UK

## Abstract

**Background:**

The descriptive information now available for primary care in the UK is unique in international terms. Under the 'Quality and Outcomes Framework' (QOF), data for 147 performance indicators are available for each general practice. We aimed to determine the relationship between the quality of primary care, as judged by the total QOF score, social deprivation and practice characteristics.

**Methods:**

We obtained QOF data for each practice in England and linked these with census derived data (deprivation indices and proportion of patients born in a developing country). Characteristics of practices were also obtained. QOF and census data were available for 8480 practices.

**Results:**

The median QOF score was 999.7 out of a possible maximum of 1050 points. Three characteristics were independently associated with higher QOF scores: training practices, group practices and practices in less socially deprived areas. In a regression model, these three factors explained 14.6% of the variation in QOF score. Higher list sizes per GP, turnover of registered patients, chronic disease prevalence, proportions of elderly patients or patients born in a developing country did not contribute to lower QOF scores in the final model.

**Conclusion:**

Socially deprived areas experience a lower quality of primary care, as judged by QOF scores. Social deprivation itself is an independent predictor of lower quality. Training and group practices are independent predictors of higher quality but these types of practices are less well represented in socially deprived areas.

## Background

Prior to 2005, few measures of quality of care were routinely available in UK primary care. Routine data were only available for services attracting a separate fee such as cervical smear rates, vaccination rates, child health surveillance, minor surgery and contraceptive services while studies collecting more detailed information data had been restricted to a limited number of volunteer practices. From 2004, however, a new system of reimbursement linked to performance indicators (the 'Quality and Outcome Framework' (QOF)) made available a rich new vein of measures of quality of care [1]. The detail and breadth of descriptive information now available for general practice in Britain is unique in international terms and makes the UK a leader in international quality improvement initiatives in primary care [2].

The Quality and Outcomes Framework is comprised of 146 indicators drawn from the following domains: chronic disease management (76 indicators covering eleven chronic diseases), 'practice organisation' (56 indicators), 'patient experience' (4 indicators) and 'additional services' (10 indicators); there is an additional 'bonus' indicator, 'access' (1 indicator) [3]. Each indicator is weighted, contributing to an overall maximum quality score for each practice of 1050 points. The performance of individual practices in England has been made publicly available.

Variability in the quality of care offered by different practices has been a cause of concern for many decades. In part it might be explained by the difficulty of providing good quality care to needy populations and in part by the intrinsic differences in the care offered. Thus when Tudor Hart described the 'inverse care law' some 30 years ago (which argued that the provision of health care was inversely proportional to the health needs of the population), his evidence from general practice was the relative newness of practice buildings in more affluent areas [4]. More detailed information about the quality of primary care provides an opportunity to revisit Tudor Hart's thesis in more depth. For example, a link between social deprivation and poorer quality of care as judged by QOF scores might arise because GPs in more deprived areas have larger, more unmanageable lists, or because they have a higher turnover of patients making it difficult to accumulate sufficient clinical successes. In other words, is there an accumulation of factors, clustering together, that hamper the delivery of high quality care in deprived areas?

Overall QOF scores have already been found to be lower in areas of higher social deprivation [5]. However, the earlier survey did not explore possible confounding by other practice variables which may have borne a stronger predictive relationship with the total QOF score. We therefore aimed to gather a broad series of nationally available practice variables in order to explore whether the relationship between overall QOF scores and deprivation remained, allowing for the effect of confounding.

## Method

### Quality and Outcomes Framework data

We obtained QOF data for all general practices in England from the Health and Social Care Information Centre, Leeds. Data for each of the domains within QOF were analysed collectively and individually: clinical, practice organisation, patient experience, additional services and access.

The QOF dataset also provided raw prevalence data for each of the eleven chronic diseases in QOF and the size of each chronic disease register enabled the proportion of 'exception reporting' to be calculated. 'Exception reporting' is the means by which certain patients are exempted from the requirements of clinical quality indicators on the basis of 'unsuitability'. For example, a patient might be exempted from the requirement to achieve a blood pressure indicator if they were unable to tolerate any additional hypotensive medication. There has been concern that some practices could use unduly high levels of 'exception reporting' as a means of achieving higher QOF scores [2].

### Practice characteristics

A detailed summary of practice characteristics was obtained from the Manchester Primary Care Research and Development Centre, University of Manchester. We obtained data on the following variables: practice list size, age/sex breakdown of registered population, number of full time equivalent GPs, training practice status, Personal Medical Services [6] or General Medical Services [6] status.

The practice list turnover was calculated as the number of new patients joining the practice list over the year April 2004 to March 2005 (minus births) divided by the number of registered patients in March 2005. Data were obtained from the National Health Applications & Infrastructure Services Programme (formerly known as the 'Exeter system') which houses registration data for all general practices in England.

### Census based variables

Based on the practice postcode, a list of Lower Layer Super Output Areas [7] was obtained for all practices. These are geographical, 'socially homogenous' areas which are arguably a better link to social measures than political units such as local government Wards. They contain an average population of around 1500. Super Output Areas form the basis of the Index of Multiple Deprivation (IMD), the most wide-ranging and up-to-date of the deprivation indices [8]. The IMD 2004 contains seven domains: income deprivation, employment deprivation, health deprivation and disability, education skills and training deprivation, barriers to housing and services, crime, living environment deprivation. Most of the data were derived from the 2001 national census but some variables such as education, crime and barriers to services are more recent. Data based on Super Output Areas were obtained, in total, for three deprivation indices: Index of Multiple Deprivation (IMD 2004), Townsend score [9] and Carstairs index [10]. Matching of practices with Super Output Areas was based on the main surgery site for each practice rather on smaller branch surgeries.

None of the above indicators includes information about ethnic minorities and place of birth, even though such data were elicited in the 2001 national census. We considered that practices serving areas where a high proportion of patients were born abroad might find it hard to achieve high QOF scores. We therefore obtained a figure for each practice, based on the Super Output Area, estimating the proportion of the resident population who were born in a developing country (born outside Europe, Australasia or North America). These data were provided by The Informatics Collaboratory Of the Social Sciences (ICOSS), University of Sheffield and derived from national census area statistics.

### Analysis methods

A dataset was constructed containing data from all 8576 practices in England, their QOF data, practice and census based variables. Data were omitted from 61 practices that were no longer independent at the end of the study year or had a list size of under 750 patients or under 500 patients per full time equivalent GP on the grounds that these were likely to be newly formed or about to be closed. The final QOF dataset contained information on 8515 practices. Postcode anomalies meant that IMD data could only be matched to 8480 practices. Disease prevalence data were available for 8430 of these practices.

Firstly, we explored the association between QOF scores and social deprivation based on the three deprivation indices using Spearman's rho for correlation testing. We used Mann Whitney U test to explore the relation between social deprivation and categorical variables. Using linear regression analysis, we then estimated univariate associations between QOF scores and other possible predictor variables. Finally, we conducted a multivariate analysis to determine the inter-relationship of several possible predictor variables. In particular, this technique would enable us to explore the relative importance of social deprivation compared to other practice or patient variables that might influence the total QOF score.

Linear regression techniques require the predictor variables to bear a linear relationship to the dependent variable. In many instances, predictor variables such as practice list size per full-time equivalent GP, were not linearly related to the total QOF score. We therefore treated list size as a categorical variable. Similarly, list turnover and the proportion of the population born abroad were converted to categorical variables. This technique requires the creation of reference group (or 'dummy' variables), which are excluded from the regression analysis. In each instance, the categorical variable containing the mean value was selected as the reference group. Thus other groups were compared with a group approximating to the national mean value for each variable.

Linear regression techniques ideally require the dependent variable to be normally distributed. The distribution of total QOF scores was negatively skewed but by performing a logit transformation the QOF score was refashioned into a normally distributed variable [11]. The regression model was constructed separately with both the unadjusted QOF score and transformed QOF score. We used forward regression models, aiming at maximising the predictive power. Variables were omitted with a P value of 0.05 or above. All calculations were conducted using the statistical packages, SPSS 13.0.

### Ethical approval

Ethical approval was obtained from the Local Research Ethics Committee (Guy's Research Ethics Committee, Chairman's action, 8^th ^February 2006). All data used in this study were publicly available.

## Results

### QOF scores and deprivation

QOF scores ranged from 0 to the maximum possible score of 1,050. Scores were highly skewed towards the high end with a median of 999.7 points and a mean of 961.8 points.

Social deprivation was inversely related to the total QOF score. Correlation coefficients with QOF scores for all three deprivation indices included in the study were almost identical (and all were significant, P < 0.001): Spearman's rho of -0.256 for the IMD, -0.261 for the Townsend index and -0.275 for the Carstairs index. Because all three indices bore almost identical relationships to the QOF score, further analysis was only conducted on the most comprehensive measure, the IMD score.

In practices located in the highest quintile of deprivation, the median QOF score was 972.3 whereas for those in the least deprived quintile, the median score was 1016.7.

### QOF scores, deprivation and other predictor variables

The results of the univariate and multivariate regression models are presented in Table 1. The model contained nine variables and explained 16.0% of the variation (adjusted r^2^) in the total QOF score (n = 8162). Missing practice data for individual variables account for the shortfall between the total number of practices in the final dataset and those included in the final model.

**Table 1:** Regression coefficients of total QOF score on practice and census based variables

**Characteristic**	**Mean (SD) or %**	**Univariate model: unadjusted regression coefficient, *B*, and 95%CI**	**Multivariate model: adjusted regression coefficient, *B*, and 95%CI**	**Multivariate model: adjusted and standardised regression coefficient, Beta.**
IMD score	25.88 (17.0)	-1.42 (-1.56 to -1.28) ***	-0.70 (-0.83 to -0.58) ***	-0.12
Group practice *versus *singlehanded practice	74.2%	76.1 (71.1 to 81.0) ***	40.3 (34.4 to 46.1) ***	0.17
Training practice	27%	60.4 (55.6 to 65.3) ***	29.7 (24.5 to 34.9) ***	0.13
PMS practice	42%	0.1 (-4.8 to 4.9)	Not included	Not included
Full time equivalent (FTE) GPs	3.10 (2.05)	15.5 (14.5 to 16.6) ***	4.4 (3.1 to 5.8) ***	0.09
List size per FTE GP:	2199 (755) (Median, 2024)			
• < 1000		-71.5 (-108.5 to -34.5) ***	Not included	Not included
• 1000–1500		-2.6 (-12.0 to 6.9)	-14.0 (-21.9 to -6.0) **	-0.04
• 1500–2000		37.6 (32.7 to 42.4) ***	Not included	Not included
• 2000–2500 ^†^		8.1 (2.9 to 13.3) **	^†^	^†^
• 2500–3000		-26.0 (-33.5 to -18.4) ***	-8.3 (-14.8 to -1.9) *	-0.03
• 3000–3500		-22.2 (-32.0 to – 11.5) ***	Not included	Not included
• > 3500		-34.6 (-45.6 to -23.5) ***	Not included	Not included
Proportion of practice list aged ≥ 75 years	7.2% (2.9%)	4.7 (3.9 to 5.4) ***	Not included	Not included
List turnover:				
• < 5%	8.4% (5.9%)	-19.1 (-24.9 to -13.2) ***	-16.4 (-21.4 to -11.4) ***	-0.07
• 5–10% ^†^		37.1 (32.4 to 41.8) ***	^†^	^†^
• 10–20%		-6.2 (-12.1 to -0.2) *	Not included	Not included
• > 20%		-40.2 (-53.8 to -26.6) ***	Not included	Not included
% born in developing country:	7.3% (9.9%)			
• < 5%		33.9 (29.0 to 38.9) ***	9.7 (3.3 to 16.1) **	0.05
• 5–10% ^†^		-4.1 (-11.6 to 3.5)	^†^	^†^
• > 10%		-40.4 (-45.9 to -34.8) ***	-8.0 (-15.4 to -0.7) *	Not included

* P < 0.05; ** P < 0.01; *** P < 0.001

^† ^Denotes the reference group which contains the national mean value and is excluded from the equation

Unadjusted and adjusted regression coefficients are presented in Table 1. Thus, for example, a group practice has, on average, 76.1 more QOF points than a singlehanded practice (unadjusted coefficient, Table 1). However, one of the advantages of group practices is that they are more likely to be training practices; removing the effect of this and other confounding variables reduces the effect of a group practice to 40.3 QOF points (adjusted coefficient, Table 1). There was, however, no advantage in access for single-handed practices as judged by the Access indicator contained in the QOF score: 94.0% of single-handed practices and 98.2% of group practices achieved this target. Finally, the adjusted regression coefficients allow the relative influence of variables measured on different scales to be compared (e.g. continuous variables such as the IMD score and categorical variables such as the training status of the practice). A larger adjusted coefficient signifies a variable with greater predictive power for the total QOF score.

Predictor variables were then removed from the equation if they added little to the explanatory model. The most frugal model obtained contains three explanatory variables: social deprivation score, group and training practice status. By omitting six of the nine variables in the original model, this frugal model still explained 14.6% of the variation in the total QOF score.

Two of the variables in the frugal model, training practice status and group practice status, were themselves associated with social deprivation. Training practices were less likely to be located in socially deprived areas: 18.8% of practices located in the highest quintile of deprivation were training practices whereas in the least deprived quintile, 37.0% were training practices. Similarly, singlehanded practices were more common in more deprived areas: 36.8% in the highest quintile of deprivation compared to 15.5% in the lowest quintile. These associations with deprivation are highly significant: for training practice status, Mann Whitney U = 5.38, Z = -13.9, P < 0.001; for group practice status, Mann Whitney U = 5.01, Z = -16.9, P < 0.001.

The dependent variable in the frugal model was the total QOF score. However, the total QOF score consists of clinical and non-clinical domains. Are all three predictor variables in the frugal model (social deprivation, training and group practice status) also significant predictors of the clinical and non-clinical components of QOF? Further regression analysis demonstrated that all three variables were significant predictors (P < 0.001). They explained 10.7% of the variation in clinical QOF scores and 12.9% of the variation in non-clinical QOF scores.

It is possible that levels of disease prevalence or of exception reporting [12] influence the final QOF score. A composite variable was created by calculating the mean prevalence of all eleven chronic diseases per 1000 registered patients. For exception reporting, we decided to choose the mean proportion of patients who were exception reported for each of the clinical indicators based on achievement of an objective measured outcome, (namely blood pressure, serum levels of cholesterol, HbA1C, TSH or lithium, or an FEV1.0 measurement by spirometry). The composite disease prevalence score was higher in practices with higher QOF scores (Spearman's rho = 0.26; P < 0.001). The mean exception reporting rate for the selected indicators was 9.0% (SD 5.2); higher levels of exception reporting were associated with higher QOF scores (Spearman's rho = 0.13; P < 0.001). The frugal regression model was re-run with the inclusion of the new variables. Adding the composite disease prevalence variable increased the explanatory power of the regression model to 14.8% and adding the composite exception reporting increased it to 19.3%.

Finally, the frugal regression model described above was repeated using the logit transformation, normalising the total QOF score. This model contained the same three predictor variables selected in the original frugal model. It explained 15.9% of the variation in the total QOF score giving similar standardised regression coefficients to the results of the original regression analysis (results available from the authors).

## Discussion

### Summary of main findings

General practices located in areas of greater social deprivation had a lower quality of care, as measured by the QOF score, than practices in more prosperous parts of the country. This relationship to deprivation was independent of other factors restraining the quality of general practice in poorer areas.

Socially deprived areas suffered from poorer quality of primary care in two further respects. Not only was social deprivation itself related to lower QOF scores, but these areas also had fewer training practices and group practices, both types of practices which deliver higher QOF scores.

Several factors that might have been thought to further reduce the quality of care were not found to contribute to reductions in the total QOF score. For example, a high proportion of patients aged 75 years and over or a high proportion of the local population being born in a developing country did not have an independent effect on the QOF score (Table 1). We found no evidence of lower QOF scores in practices with very high list sizes (above 3500 per GP), nor, by contrast, were smaller than average list sizes of any apparent advantage in gaining a higher QOF score. In the frugal regression model list size did not feature at all as a predictor of the total QOF score. Similarly, high turnover of the registered practice population appeared to make it more difficult to achieve higher QOF scores, but once other confounding variables were taken into consideration, list turnover had very little independent effect and again, this variable did not feature in the frugal regression model. Practices coping with high chronic disease prevalence, rather than being overwhelmed and achieving lower QOF scores, reported higher QOF scores.

### Findings in relation to other studies

Previous studies of the quality of care offered in British general practices have relied on smaller selective samples and have worked with a much more limited range of performance indicators. Even so, higher quality care in group practices has been reported, which is consistent with our findings. Campbell *et al *[13] noted the multidimensional nature of quality, finding that although diabetic quality indicators were higher in larger practices, access to care was better in smaller practices. In this study, however, we found no evidence of better access for smaller practices. Our findings are at odds with a smaller earlier study of practices which found that the quality of care in group practices was no better than in singlehanded practices although the only clinical indicators measured were cervical smear and vaccination targets [14]. Chronic disease management is increasingly complex and just as complex patients need longer consultations [13] it appears that they also benefit from care provided by the wide range of health professionals typically seen in larger practices.

The development of the UK QOF has taken place within the context of international attempts to improve the quality of primary care. Realising the importance of providing a strong operational setting for delivery good quality care, Engels *et al *[15] have developed the European Practice Assessment instrument, tested in nine countries and consisting of 57 validated measures of the quality of practice management. Some initiatives have required the development of an assessment process involving practice based visits such as the 'Visit Instrument to assess Practice management (VIP), widely used in Holland [16]. The Organisation for Economic Co-operation and Development (OECD) has developed a Healthcare Quality Indicator (HCQI) consisting of clinical and organisational domains, emphasising the public health aspects of primary care, which will be applicable to both developing and developed countries [17]. Many quality improvement instruments (including QOF) have not included direct patient input. International studies have shown little relationship between objective measures of quality and patient satisfaction [18]. In the case of single-handed practices, an inverse relationship between patient satisfaction and practice based quality measures has been demonstrated [16]. This discrepancy reflects a tension between professional models of assessment and attempts to determine patients' perspectives.

Our findings contribute to the literature about the effect of social deprivation on the quality of health care [19-22] though it is not clear how a deprived population leads to compromises in the quality of health care. Overwhelming clinical demand from a high morbidity population might seem a likely factor but we found the opposite: practices achieving higher QOF scores reported higher overall prevalence of the chronic diseases represented in QOF. Estimates of morbidity may, however, have been distorted by inadequate recording since prevalence measures are derived from data reported in the practice disease register. The validity of these disease prevalence data has not been tested [23] Recording bias may have resulted in unduly low estimates of disease prevalence in deprived areas. Conversely, some practices may have been highly efficient at computerised coding of chronic disease registers and QOF indicators, thus boosting their reported achievement in both activities. Variations in the efficiency with which practices coded clinical and managerial data may have introduced another variable which we were unable to measure and which could have influenced conclusions drawn from our final regression model.

Training practices had higher QOF scores than non-training practices. These practices are 'accredited' for the purposes of postgraduate training of GP Registrars. Many of the quality indicators contained within QOF are part of the three year inspection cycle required of all UK training practices giving training practices a head start in terms of QOF achievements [24]. The importance of education and training in organisations wishing to develop high quality services has already been described and may have contributed to an ethos within the practice resulting in the delivery of high quality care [25].

### Limitations of the present study

An important limitation of the study was that the deprivation score was assigned on the basis of the practice's geographical situation with an assumption that practices serving poorer populations are situated in their midst. For various reasons GPs may choose to practice in a less deprived area than the main populations they serve but this is likely to bias results towards under- rather than over-estimating the relationship between deprivation and quality of services.

A further limitation is the use of publicly available descriptive data for general practices. It is quite possible that other variables, if available, could have had a strong predictive effect on the total QOF score. For example, Sutton & McLean [26] were able to obtain details of practice funding in one Scottish health authority (Ayrshire and Arran) and found a significant relationship between higher funding and lower total QOF score, independent of confounding by deprivation. Such information is not nationally available for practices in England.

The regression equations presented in Table 1 should be treated with some caution. Total QOF scores were skewed meaning that outliers (low scorers) would have a disproportionate effect on the predictive power of the model. However, the more cautious logit transformation probably provides a more reliable estimate of the combined effects of each predictor variable and when this was used, it resulted in a slightly higher predictive power. This model has not been presented in detail because, by transforming the dependent variable, the regression coefficients no longer provide direct information about the influence of predictor variables on the QOF score.

There is an assumption in this study that total QOF score really does indicate the quality of care delivered by practices in England. Individual indicators were carefully selected with an evidence base of literature to support their inclusion in the QOF [2], though large areas of personal care provided by the general practitioner were omitted. Nevertheless, in the round, the QOF is a broadly based and objective measure of items recommended by both the profession and patients. As such, it is likely to provide a good snapshot of quality.

### Implications and future research

The new contract for general practitioners specifically excluded any 'compensation' for working in socially deprived areas, a situation likely to be exacerbated by the funding penalty of lower QOF scores. The 'inverse care law' continues to operate in modern primary care but can that be corrected, in part at least, by promoting the group and training practices known to be associated with higher quality of care?

Exception reporting may also be contentious [2,27]. Although strict criteria for exception reporting have been defined and practices can expect close scrutiny if their exception reporting level appears high [27], we found wide variation in levels of exception reporting but our final regression model was only modestly influenced by the level of exception reporting. Doran *et al *[12] found that exception reporting rates were 'not extensive' with a median rate of 6%. On the other hand, some specific indicators have much higher rates of exception reporting [28]. Further study is needed on the contribution of exception reporting to achieving higher QOF scores.

The total QOF score is itself a composite indicator. We pursued our analysis of predictor variables, confirming that training practice status, group practice status and lower social deprivation remained significant predictors of both the clinical and the non-clinical components of QOF. Although others have used this methodology, analysing the total QOF score as a single indicator [5], further work is needed to determine which individual components of QOF are maximised in training practices, group practices and practices in less deprived areas. Measures to address inequalities of care can only be implemented when more information is available on the specific shortfalls in individual quality indicators.

Quality of care varies very widely indeed but the variables that we were able to measure only explained a small proportion of that variation. Further study is needed to determine other factors which might influence quality. It is likely that these other factors are linked to the values of the organisation and the attitudes of practice managers, clinical staff and the wider primary care team and are more likely to be amenable to exploration through qualitative studies.

## Conclusion

Providing good quality care to deprived populations continues to be challenging. Not only is social deprivation itself an independent predictor of lower QOF scores but both training and group practices, which are characterised by higher QOF scores, are less well represented in deprived areas.

## Competing interests

The author(s) declare that they have no competing interests.

## Authors' contributions

MA and DA designed the study and jointly carried out the statistical analysis. MA prepared the first draft of the paper which was extensively revised by both authors. The final version was read and approved by both authors.

## Pre-publication history

The pre-publication history for this paper can be accessed here:


